# A study on sexual development of SD rats by using KiSS1RNA interference mediated by lentivirus-based vectors

**DOI:** 10.1186/1687-9856-2013-S1-O28

**Published:** 2013-10-03

**Authors:** Dong Hong, Li Pin

**Affiliations:** 1 Departmentment of Endocrinology, Chirdren’s Hospital of Shanghai , Shanghai Jiaotong University, Shanghai, 200040, China

## 

To explore the possible mechanism of KiSS1 in control GnRH secretion participate in sexual development onset and normal reproduction regulation by investigate the changes of expression of KiSS1, GnRH in hypothalamus and LH, FSH, E2 in serum,by using RNA interference Mediated with Lentivirus-based Vectors, after Interfering expression of KiSS1.

Constructed the eukaryotic expression plasmid of rat KiSS1 gene and four plasmid expressing KiSS1 microRNA, then respectively co-transfected them into 293T cells. Real-time PCR detected the expression of KiSS1 mRMA in order to filter the most effective microRNA plasmid.Constructed recombinant entivirus and determined the titer, then they were intracerebroventricularly infused into the brain of Sprague-Dawley rats (21-day-old) . The three groups included interference virus group, Lentivirus-control,NS-control, Ten rats in each group animals were sacrificed at 30-day-old, 35-day-old, 45-day-old. Then the expression of KiSS1 and GnRH mRNA were conducted in the rat hypothalamus with Real-time PCR, the LH, FSH, E2 in serum were examined with Chemiluminescence method. HE staining was used to observe histomorphology ofovarian.

Successfully filtered the most effective microRNA plasmid and constructed recombinant lentivirus. The titer of recombinant lentivirus was 8×10^8^TU/ml. The level of KiSS1 mRNA in interference virus group was significantly reduced after infecting recombinant lentivirus compared with control group at 30d NS-control group0.2±0.02, Lentivirus-control group 0.188±0.023, interference virus group 0.106±0.018; at35d NS-control group 0.433±0.046; Lentivirus-control group 0.41±0.034; interference virus group 0.218±0.025; at 45d NS-control group 0.315±0.048; Lentivirus-control group 0.282±0.052; interference virus group 0.215±0.033, at 30d F=112.40 P<0.01; at 35d, F=209.5 P<0.01; at 45d, F=5.2, P<0.05. The level of GnRH mRNA in interference virus group was significantly reduced after infecting recombinant lentivirus compared with control group (at 30d NS-control group 0.387±0.044; Lentivirus-control group 0.373±0.05; interference virus group 0.23±0.03; at35d NS-control group 0.517±0.048; Lentivirus-control group 0.53±0.052; interference virus group 0.407±0.03; at 45d NS-control group 0.468±0.03, Lentivirus-control group 0.479±0.038; interference virus group 0.455±0.054,at 30d,F=24.6, P<0.01at35d,F=20.90 P<0.01). The difference was statistical significance. The level of LH in interference virus group is lower than other two groups at 35d (NS-control group 0.111±0.008 mU/ml ; Lentivirus-control group 0.106±0.006mU/ml; interference virus group 0.101±0.004mU/m F=5.98 P<0.05). The level of FSH in interference virus group is lower than other two groups at 35d (NS-control group 0.219±0.015mU/ml; Lentivirus-control group 0.215±0.014mU/ml; interference virus group 0.205±0.014mU/ml F=9.72 P<0.05). The level of E2 in interference virus group is lower than other two groups(at 35d, NS-control group 56.04±4pg/ml; Lentivirus-control group 52.9±3.26pg/ml; interference virus group 46.8±3.01pg/ml; at45d NS-control group 57.4±5.5pg/ml; Lentivirus-control group 58.1±3.02pg/ml; interference virus group 52.4±3.57pg/ml; at 35d,F=25.25 P<0.01; at 45d,F=7.63 P<0.05). Meanwhile, the time of vaginal orifice opening of the interference virus group was significantly later than control virus group and normal saline group (the interference virus group37.4±1.57d, control virus group 35.2±1.19d, and normal saline group 34.9±0.99d, F=18.1 P<0.01). The histology of ovary showed similar results.

Our result show that the lentiviral vector of KiSS1-microRNA can inhibited the expression of KiSS1 stabily, efficiently and specificly. Lentiviral with KiSS1-microRNA can affect the expression of GnRH and The levels of sex hormone. Intralateroventricular microinjection of KiSS1-microRNA Lentiviral can delay sexual development of SD female rat.

Lentivirus-mediated RNAi can effectively suppress the expression of KiSS1 stabily,as a result the expression of GnRH gene can be suppressed, then affecting sexual development. It may provide a potential tool for the study of targeting control of sexual development in vivo and treating precocious puberty and other diseases.

